# Characteristics of oral health of patients with X-linked hypophosphatemia: case reports and literature review

**DOI:** 10.1038/s41405-024-00223-6

**Published:** 2024-05-31

**Authors:** Ana Arhar, Alenka Pavlič, Luka Hočevar

**Affiliations:** 1 Department of Paediatric and Preventive Dentistry, Faculty of Medicine, University of Ljubljana, Vrazov trg 2, Ljubljana, Slovenia; 2 Department of Paediatric and Preventive Dentistry, University Medical Centre Ljubljana, Zaloška 2, Ljubljana, Slovenia

**Keywords:** Oral diseases, Diseases

## Abstract

**Background:**

Oral health is impaired in X-linked hypophosphatemia (XLH), resulting in delayed dental development, malocclusion, and radiographic abnormalities. This study investigates the oral manifestations in Slovenian XLH patients, focusing on enamel and dentin abnormalities and a literature review of spontaneous periapical abscesses in XLH cases.

**Objectives:**

To report XLH patients with specific oral signs and symptoms, histological analysis of affected teeth, and review of reported cases of XLH patients with spontaneous periapical abscesses.

**Methods:**

*Case reports*: Seven XLH patients from the National Registry of Patients with Rare Diseases underwent a detailed oral examination, including X-ray reviews. The patients who were expected to have tooth exfoliation or extraction were asked to donate their teeth for histological analysis by scanning electron microscopy. *Literature search*: A literature search of four electronic databases and a manual bibliography search aimed to identify documented cases of XLH with periapical abscesses up to January 21, 2024. Inclusion criteria were confirmed XLH patients with periapical abscesses in English peer-reviewed publications.

**Results:**

Tooth samples from three XLH patients showed reduced dentin mineralisation, affecting one-third to one-half of the outer dentin. Inadequate mineralisation, uneven dentin tubules, and cracks and chipping in the enamel were observed, indicating mineralisation deviations. Similar cracks extended into the dentin and were also present in the root of the examined tooth. Based on the content of the 75 items identified in the search, spontaneous abscesses are not uncommon in patients with XLH.

**Conclusions:**

XLH significantly affects patients’ lives and requires lifelong treatment. Dental examinations consistently revealed oral problems, including malocclusion. Histological analysis confirmed structural changes, especially in the dentin. Despite continued treatment, XLH patients may have an increased risk of oral pathologies. Further research is needed to understand the impact of XLH and its treatment on dental health.

## Introduction

X-linked hypophosphatemia (XLH), also known as familial hypophosphatemic vitamin D-resistant rickets, is a rare disease with a prevalence of 1.7 to 4.8 per 100,000 people (children and adults) [1]. Several signs and symptoms characterise this disease, particularly in the skeleton (osteomalacia) and teeth (odontomalacia) [1, 2]. The diagnosis of XLH is based on clinical, radiological and biochemical findings and confirmed mutation in the *PHEX* gene. Genetic analysis is recommended, particularly in patients with a negative family history [1].

The disease develops due to mutations in the *PHEX* gene located on the X chromosome [3], resulting in an inactive form of the phosphate-regulating endopeptidase homologue [4]. This enzyme affects small integrin-binding ligand, N-linked glycoproteins (SIBLING proteins) and fibroblast growth factor 23 (FGF23), which in turn increases phosphate excretion and inhibits bone and tooth mineralization [4].

In bone, an inactive or absent phosphate-regulating enzyme triggers an increased release of FGF23 into the serum. Elevated FGF23 in the serum impacts the kidneys, intestines, bones, muscles, teeth and other tissues [5]. In the kidney, where FGF23 influences the downregulation of renal tubular phosphate transporters, an increased level of FGF23 leads to increased phosphate excretion. In the intestine, the absorption of phosphates is reduced due to weakened activation of calcitriol (i.e. vitamin D) and inhibited secretion of parathyroid hormone (PTH), both of which are also influenced by elevated FGF23 [4]. Elevated FGF23 in serum indirectly limits renal calcitriol synthesis and increases its degradation. Consequently, calcitriol cannot compensate for hypophosphatemia.

Reduced serum phosphate levels lead to impaired mineralisation and bone growth, disproportionate short stature, bowing of the legs, osteoarthritis, pseudofractures, musculoskeletal pain, enthesopathies, fatigue and hearing impairment [1, 4, 5]. Due to premature fusion of the cranial sutures during development, patients may have an abnormal skull shape characterised by flattening of the parietal bone, frontal bossing and widened sutures. Their head is longer than expected compared to its width (i.e. dolichocephaly) [1]. These patients may also develop central nervous system complications associated with Arnold-Chiari malformations [1, 4, 6–9]. The symptoms of XLH show tremendous variability between patients due to the degree of hypophosphatemia, age at onset of systemic treatment and other factors [10]. It should also be noted that patients with XLH, regardless of their age, report a severely impaired quality of life due to the symptoms of the disease [5, 11].

Oral health is also significantly impaired in XLH patients. In children, dental development is typically delayed, and eruption patterns are abnormal. Certain dental malocclusions are more common, such as an open bite, maxillary retrognathism and impacted or ectopic maxillary canines [12–14]. Nevertheless, there are few reports of orthodontic treatment of patients with XLH [15]. Radiographic examinations of deciduous and permanent teeth reveal shorter roots, root dysplasia, enlarged pulp chambers and high pulp horns extending to the dentin-enamel junction (DEJ) [16]. Some authors reported taurodontic permanent molars [17–19]. Thin trabeculae of the jaw bones and the absence of lamina dura around the tooth roots have also been noted [10, 20–22].

Histological findings confirm dental aberrations in patients with XLH [23]. The circumpulpal dentin exhibits marked globular dentin and an increased width of the predentin [22]. The formation of large interglobular spaces filled with a non-mineralised organic matrix is due to the inability of the calcospherites to fuse [10, 14, 24–27]. The roots of the teeth are covered by a thinner layer of unevenly mineralised dental cementum [28, 29]. The enamel may also be less mineralised, with an irregular surface and areas of extensive microcracks or crater-shaped depressions [22, 23]. For this reason, dyschomic changes in the enamel are visible in some teeth [20].

Given all this, it is not surprising that bacteria can easily penetrate through anatomically and histologically defective enamel and dentin without significantly decomposing the hard dental tissue. Spontaneous periapical infections of non-carious teeth and the absence of a history of trauma or fracture are common oral findings in patients with XLH [24]. The abscesses may sometimes spread to the surrounding anatomical structures and cause maxillofacial cellulitis [1]. In addition, poorer oral health increases the incidence of developing periodontitis. In particular, adults with undiagnosed XLH often experience severe periodontal loss with profound damage to the cementum, periodontal ligament and alveolar bone [28, 30].

The primary aim of this study is to determine the oral manifestations in individuals diagnosed with XLH from the Slovenian national registry. The study describes the oral signs and symptoms observed in XLH patients, with particular attention to abnormalities in the histological structure and mineralisation of enamel and dentin in both deciduous and supernumerary teeth of the included patients. In addition, a detailed literature review of XLH cases with spontaneous periapical abscess formation, a common clinical finding in patients with XLH, was performed.

## Materials and methods

### Patients and oral clinical examination

As part of a comprehensive medical study of children and adolescents with XLH, all patients enroled in the National Registry of Rare Diseases Patients with confirmed XLH underwent a thorough oral clinical examination. Patients were also asked to bring their previously obtained radiographs. All patients in whom physiological exfoliation of a deciduous tooth was expected, or tooth extraction was indicated were asked to donate the tooth for histological analysis.

Before the clinical examination, the patients and their parents were informed in detail about the aim of the study and their voluntary participation. All subjects who participated in the study or their parents gave their written consent to participate. The study was conducted in full compliance with the Declaration of Helsinki, and the research protocol was approved by the Medical Ethics Committee of the Republic of Slovenia (Act No. 0120-546/2023/3).

### Scanning electron microscopy

The XLH patients were asked to donate their physiologically exfoliated deciduous teeth and teeth that were scheduled for extraction due to complications or orthodontic reasons. We advised the parents to place the exfoliated deciduous teeth in saline solution and bring them to the dental office within 48 h. All tooth samples, both those brought by the parents of XLH patients and those extracted in a dental office, were fixed in 10% neutral buffered formalin, rinsed extensively with saline and then embedded in epoxy resin (EpoFix Kit, Struers Inc., Cleveland, OH, USA). After polymerisation, the surfaces were ground to expose the axial cross-sections of the teeth. They were then polished, etched with 37% orthophosphoric acid for 30 s, rinsed with distilled water spray for 30 s, dried with compressed air, dehydrated with 70% ethanol, dried again and sputter-coated with carbon (Vacuum Evaporator, Type JEE-SS; Japan Electron Optics, Tokyo, Japan). The samples were then subjected to ultrastructural analysis using a scanning electron microscope (SEM) (Thermo scientific Quattro ESEM, Waltham, Massachusetts, USA) in secondary electron imaging (SEI) and backscattered electron (BSE) mode. The micrographs were taken at an accelerating voltage of 10 kV and a working distance of 10 mm.

### Literature search

A literature search was performed to identify all documented cases of XLH in which spontaneous periapical abscesses were reported. A comprehensive search of four electronic databases (PubMed, Embase, Web of Science and Scopus) using the following keywords: “rachitis AND abscess AND case report”, “XLH AND abscess AND case report,” and “hypophosphatemic AND abscess AND case report” was performed to identify all documented cases of XLH in which spontaneous periapical abscesses were reported (see File S1). The search was supplemented by manual searches of the bibliographies of included studies for relevant articles/case reports. We included case reports of patients with confirmed XLH who had periapical abscesses on deciduous and/or permanent teeth. The search was limited to reports published in English and in peer-reviewed journals. The last search was completed on January 21, 2024.

## Results

### Patients

All seven XLH patients (4 females and 3 males) in the national registry agreed to participate in this study (Table 1). The age of the patients ranged from 5 years 6 months to 23 years 11 months. On average, they were diagnosed with XLH at the age of 6.5 years. Patients originating from families in which other family members had already been diagnosed with XLH (patients no. 1, no. 6 and no. 7 in Table 1) were diagnosed at an average age of 1.4 years. One patient (No. 5 in Table 1) immigrated to Slovenia at the age of 12 and was diagnosed with XLH at the age of 16. In their medical history, all patients also described signs and symptoms of an insufficiently mineralised skeleton.

**Table 1:** Findings of medical and dental anamnesis, oral clinical examination, and analysis of dental X-ray images of XLH patients included in the study.

No.	Age	Gender	The age when XLH was diagnosed	Family members affected	Systemic XLH signs and symptoms	Dental history	Soft oral tissues	Teeth	Malocclusions
1.	5 years and 6 months	F	2 years and 6 months	Yes; Mother	Delayed growth, deformities of the lower extremities; Fatigue and pain in legs (before XLH was diagnosed)	Deciduous molars erupted before incisor and canine teeth; An occasional occurrence of aphthous ulcers; Dental caries	*	Vital teeth of milk glass colour; Calculus; Infraocclusion of teeth 74 and 84	Dental crowding (lower and upper anterior region); Mesial occlusion (left); Singular antagonism (right); Bilateral posterior cross-bite
2.	6 years and 7 months	F	3 years	No	Delayed growth and problems with legs (before XLH was diagnosed)	Dental caries	Fistulae of teeth 64 and 65	Milky-glass teeth colour; Hypoplastic pits on teeth 73, 83 and 31 (incisal on buccal surfaces); Infraocclusion of a tooth 74; non-vital teeth: 64, 65 and 84	Dental crowding (lower anterior region); Increased anterior overjet; Deep bite
3.	12 years	F	9 years	No	Delayed growth and orthopaedic operation (before XLH was diagnosed)	An occasional occurrence of aphthous ulcers	A remnant of a scar between teeth 22 and 23	Calculus; Hypomineralised enamel (buccal surfaces of the tooth crowns 45 and 21); Hypoplastic thinned enamel on incisal part of lower incisors	Posterior cross-bite (right); Singular antagonism (left)
4.	18 years and 7 months	F	13 years	Presumably grandmother	Unstable knees; Knee pain after a long walk; Delayed growth; Idiopathic bone fracture	*	Gingivitis	Calculus	Dental crowding (lower and upper anterior region); Bilateral posterior cross-bite; Mesial occlusion
5.	18 years and 8 months	M	16 years	No	Knee problems	Teeth 14, 24 and 26 were extracted	Gingivitis; Fistula of a tooth 16	Calculus; Localised hypoplastic defects on the upper incisor teeth; Thinned incisal edges on the lower incisor teeth; Non-vital teeth 16 and 46	Distal occlusion (right); Improper bilateral position of the teeth (upper posterior region); Bilateral posterior cross-bite
6.	18 years and 9 months	M	10 months	Yes; Mother	Deformities of the lower extremities	Recurrent aphthae on 2-3 weeks; Dental abscess in the region of tooth 36	Gingivitis; Fistula of a tooth 36; Dental abscess in the lower anterior vestibular area	Calculus; Non-vital teeth: 31, 32, 33, 41 and 36	Over-bite (8 mm)
7.	23 years and 11 months	M	12 months	Yes; Mother and grandmother	Pain in the lower extremities; Scoliosis	Teeth sensitive to cold and hot stimuli/Hypersensitive teeth; TMJ dislocation with more pronounced mouth opening; Third molars erupted the age of 11	Gingivitis; Aphthae in the vestibule near the tooth 43; Fistulae of a tooth 36	Non-vital teeth: 11, 21 and 36; A supernumerary tooth located buccally next to teeth 16 and 17	Dental crowding (lower and upper anterior region); Mesial occlusion (left); Ectopic upper left canine

*F* female, *M* male; * not specified, *TMJ* temporomandibular joint.

### Oral clinical examination

**Fig. 1:**
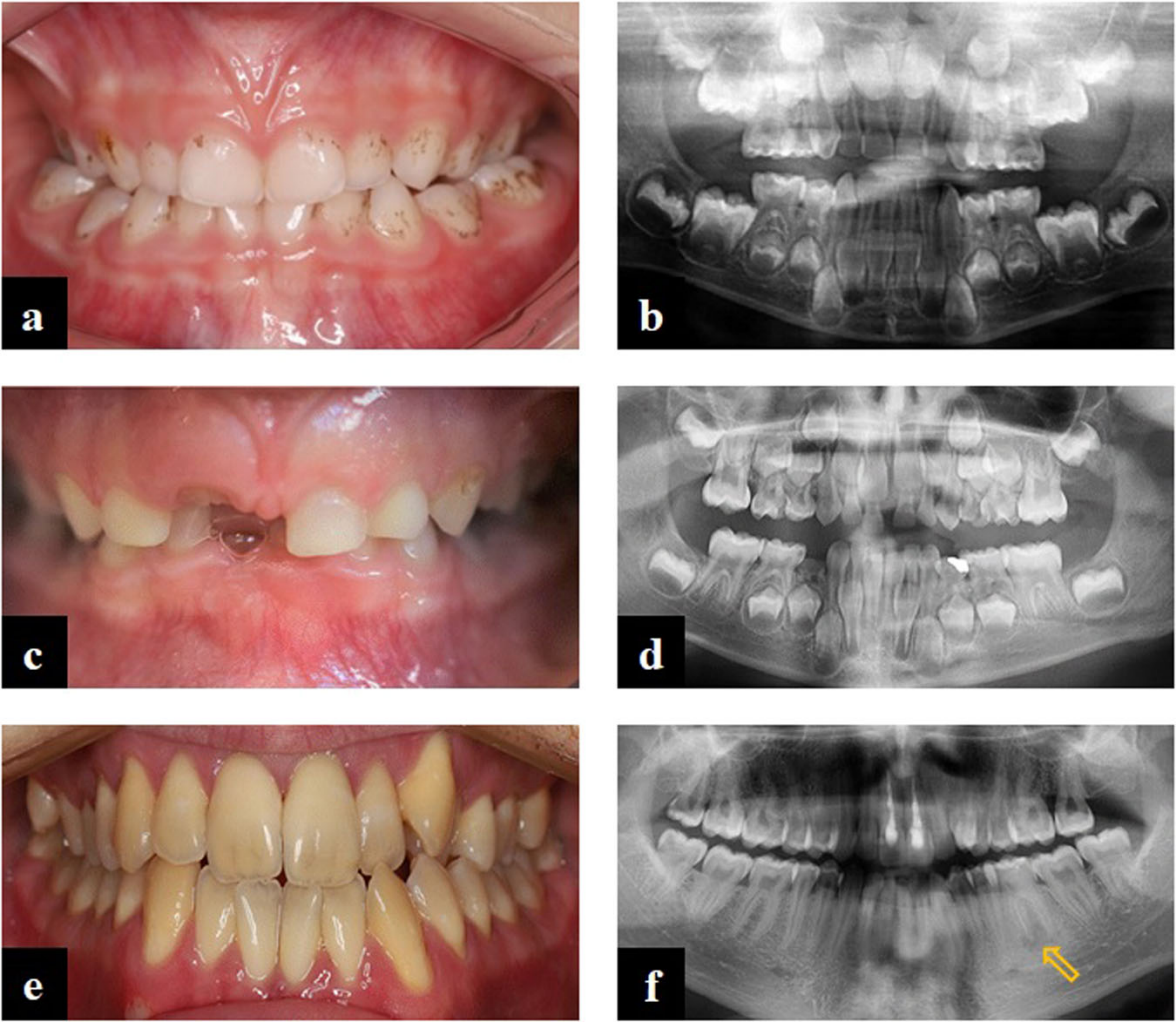
Intraoral view and panoramic radiograph of three patients with XLH. **a** The deciduous dentition of a 5.5-year-old girl diagnosed with XLH shows milk glass-coloured dental crowns lined with dental plaque and calculus. Note the bilateral cross-bite in the posterior region. The panoramic radiograph (**b**) shows the presence of all permanent tooth buds. Note the less radiopaque enamel and dentin, the unrecognisable lamina dura and the enlarged pulp chambers, especially in the deciduous molars. **c** The milk glass-coloured early mixed dentition of a 6.5-year-old female XLH patient with a deep bite and a lack of space in both dental arches. The panoramic radiograph (**d**) showed large pulp cavities and poor radiopacity of enamel and dentin of all deciduous teeth and the developing permanent teeth. **e** The permanent dentition of the almost 24-year-old patient with XLH with an ectopic position of the upper left canine and present supernumerary tooth. The panoramic radiograph (**f**) shows signs of periapical inflammation due to the progression of pulp inflammation of tooth 36 (arrow), bone resorption due to inflammation of the lower incisors and endodontic treatment of both upper central incisors.

**Fig. 2:**
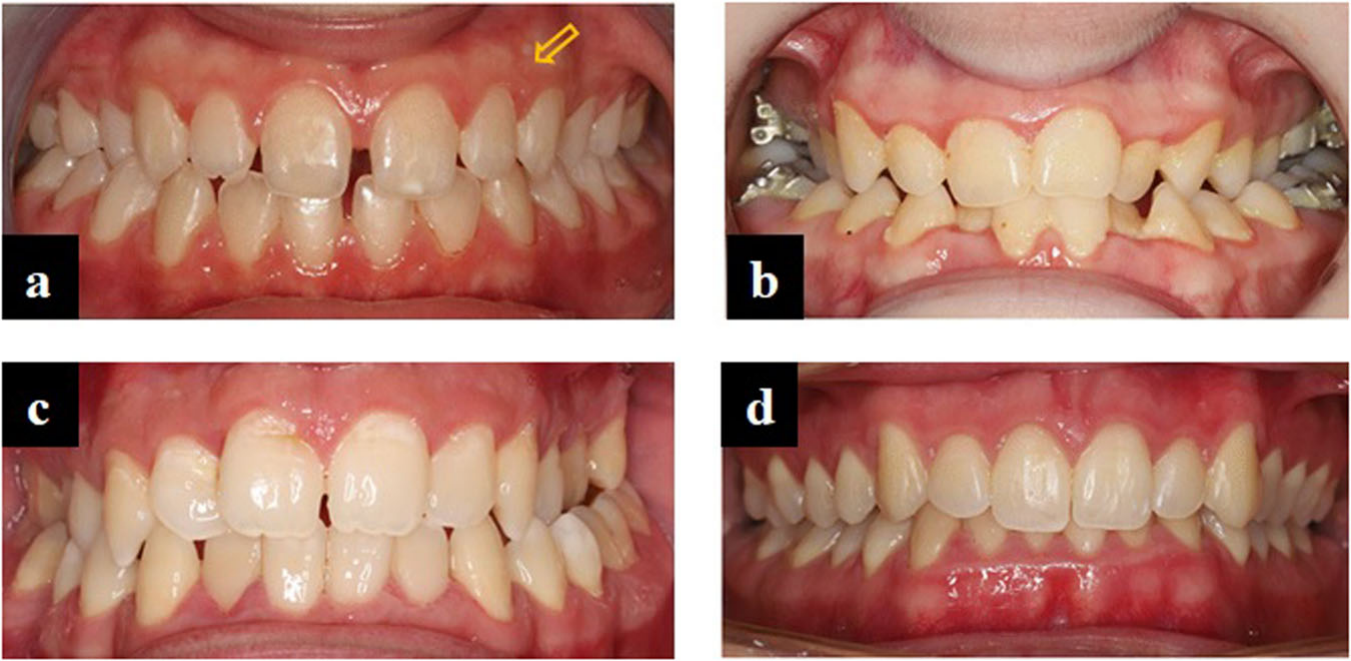
Intraoral view of four patients with XLH. **a** The permanent dentition of a 12-year-old girl suffering from XLH. All teeth are vital, dull, glassy colour with whitish areas and under orthodontic treatment for a posterior cross-bite on the right side. A scar on the mucosa between teeth 22 and 23 (arrow) is most likely the remnant of a fistula associated with a deciduous tooth. **b** The permanent dentition of a young adult XLH patient with generalised marginal gingivitis and calculus (mainly on the lingual surface of the lower anterior teeth) is also undergoing orthodontic treatment. All her teeth are vital. **c** In a young adult male XLH patient with bilateral posterior cross-bite, three permanent teeth have already been extracted (teeth 14, 24, and 26). **d** The complete permanent dentition of an 18 years and 9 months old male XLH patient has an active fistula due to spontaneous periapical inflammation of tooth 36.

Among the XLH patients, there were some significant similarities in the pathological signs and symptoms in the oral cavity (Table 1). Three of the seven patients reported frequent occurrence of aphthae. Five patients displayed calculus: the girl had calculus over the entire dentition, and the other four on the lower anterior teeth. The teeth were of milk-glass to yellowish colour, with whitish discolouration in certain areas and/or thinner enamel on the incisal edges of the teeth (Figs. 1, 2). Three of all permanent teeth had already been extracted, and nine were non-vital. Active dental fistulae associated with non-vital permanent and deciduous teeth were found in all three male patients and one female patient, respectively. The radiographs of the patients confirmed poor mineralisation of the dentin, enamel and bone (Figs. 1b, d, f). In general, the teeth showed large pulpal spaces. The radiopacity of the alveolar bone was less pronounced; as a result, some periapical processes were also slightly less visible. All XLH patients in this study had malocclusions. The most common findings were dental crowding in the dental arches, posterior cross-bite and mesial or distal occlusion. Orthodontic treatment was ongoing in some patients, and one patient discontinued treatment prematurely.

### Scanning electron microscopy

We collected dental specimens from three XLH patients: an exfoliated deciduous incisor (tooth 72) and a deciduous molar (tooth 55) extracted due to periapical inflammation from a 5.5-year-old girl, a crown fragment of a deciduous molar (tooth 65) from a 6-year and 7-month-old girl, and a supernumerary tooth from an almost 24-year-old man. One specimen (tooth 65) was first observed under the SEM as unetched. Later, the sputtered carbon layer was grounded off; the specimen was etched with 37% orthophosphoric acid and prepared for further observation under SEM as previously described.

**Fig. 3:**
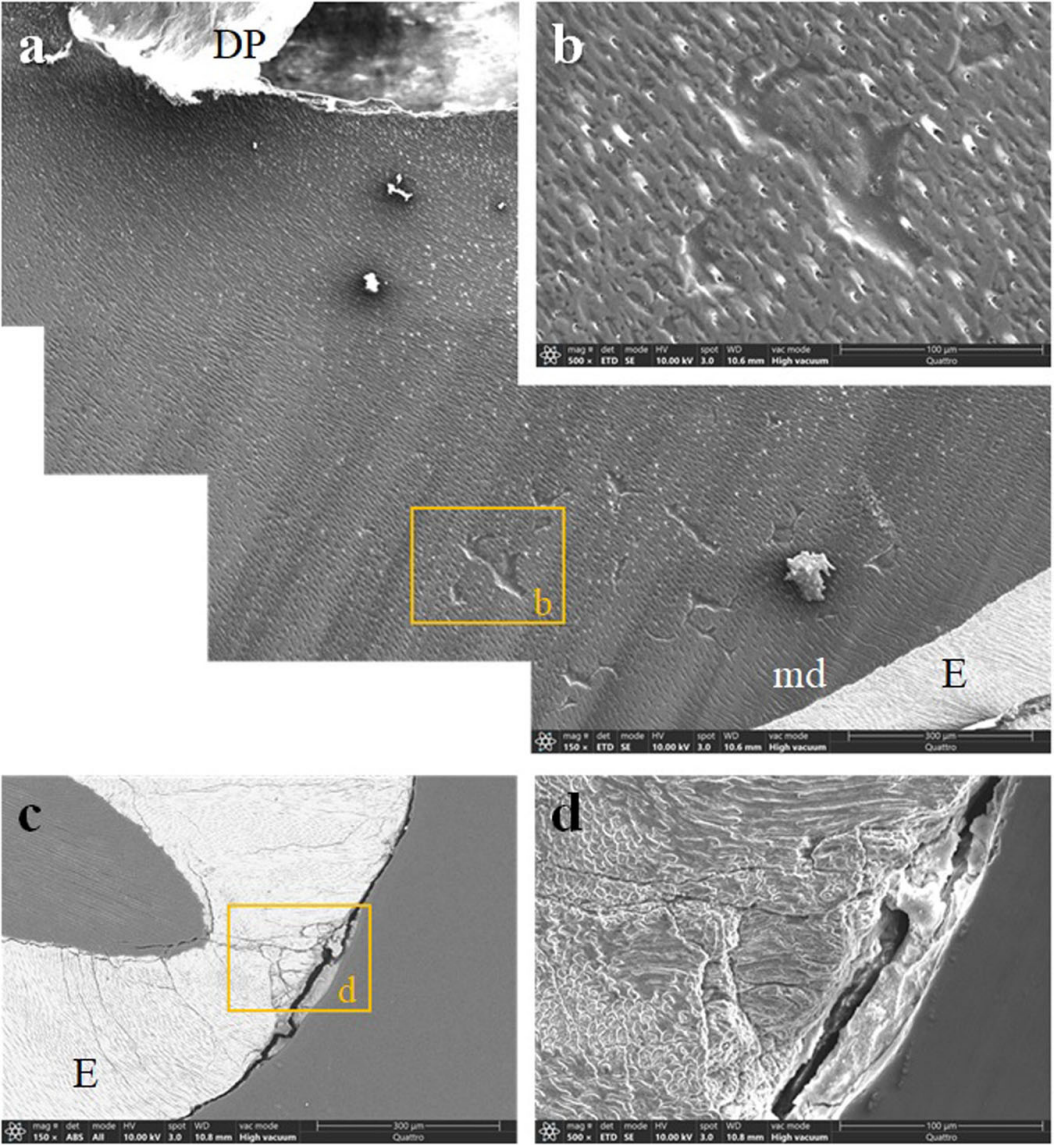
An exfoliated deciduous incisor. **a** A compound image of a longitudinally sectioned XLH-affected deciduous incisor (tooth 72) shows a narrow band of mantle dentin beneath the enamel transitioning to poorly mineralised dentin extending over the outer half of the dentin thickness (×150, SE, etched). **b** Depressions are clearly visible at higher magnification in this insufficiently mineralised dentin (×500, SE, etched). **c** The enamel is interspersed with cracks, especially at the incisal edge (×150, BSE and SE, etched), **d** even more visible at higher magnification (500x, SE, etched). BSE backscattered electrons, E enamel, DP dental pulp, md mantle dentin, SE secondary electrons.

**Fig. 4:**
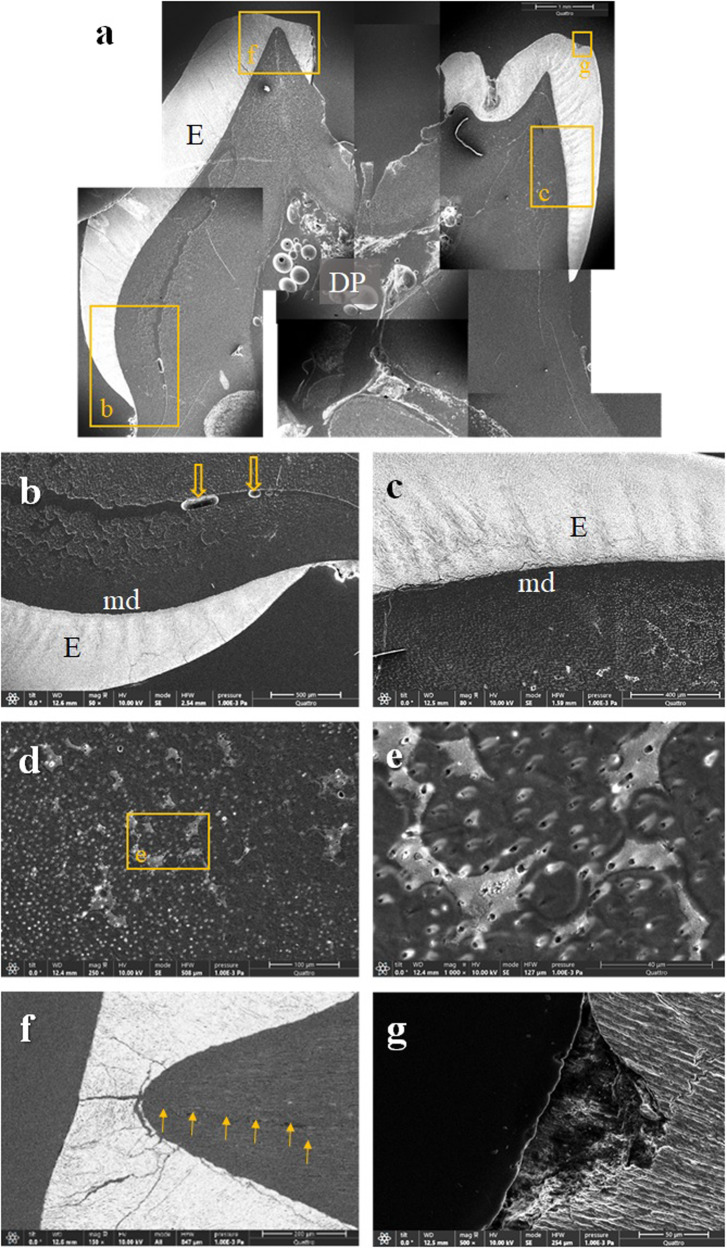
A deciduous molar extracted due to periapical inflammation. **a** A compound image of a longitudinally sectioned XLH-affected deciduous molar (tooth 55) reveals an aberrant dentin structure and some enamel anomalies. The enamel shows numerous cracks, and in the central part of the occlusal surface, the enamel is missing with an extensive cavitated lesion (×25, SE, etched). **b** On the palatal side, a thin layer of mantle dentin delimits profoundly aberrant dentin from the enamel. Note the unusual voids (arrows) in the centre of the poorly mineralised dentin and cracks in the enamel (50x, SE, etched). **c** On the buccal side, the mineralisation and structure of the dentin are also inadequate (80x, SE, etched). **d** The image shows dentin from the middle part of the root, again with defective interglobular spaces (250x, SE, etched), **e** and indentations that are more visible under higher magnification (×1000, SE, etched). **f** On the palatal cusp, the tooth enamel is riddled with cracks. The wider crack continues into the underlying dentin and extends to the bulk of the dentin (yellow arrows) (×150, BSE and SE, etched). **g** A non-carious defect is present on the surface of the buccal cusp (×500, SE, etched). BSE backscattered electrons, E enamel, DP dental pulp, md mantle dentin, SE secondary electrons.

**Fig. 5:**
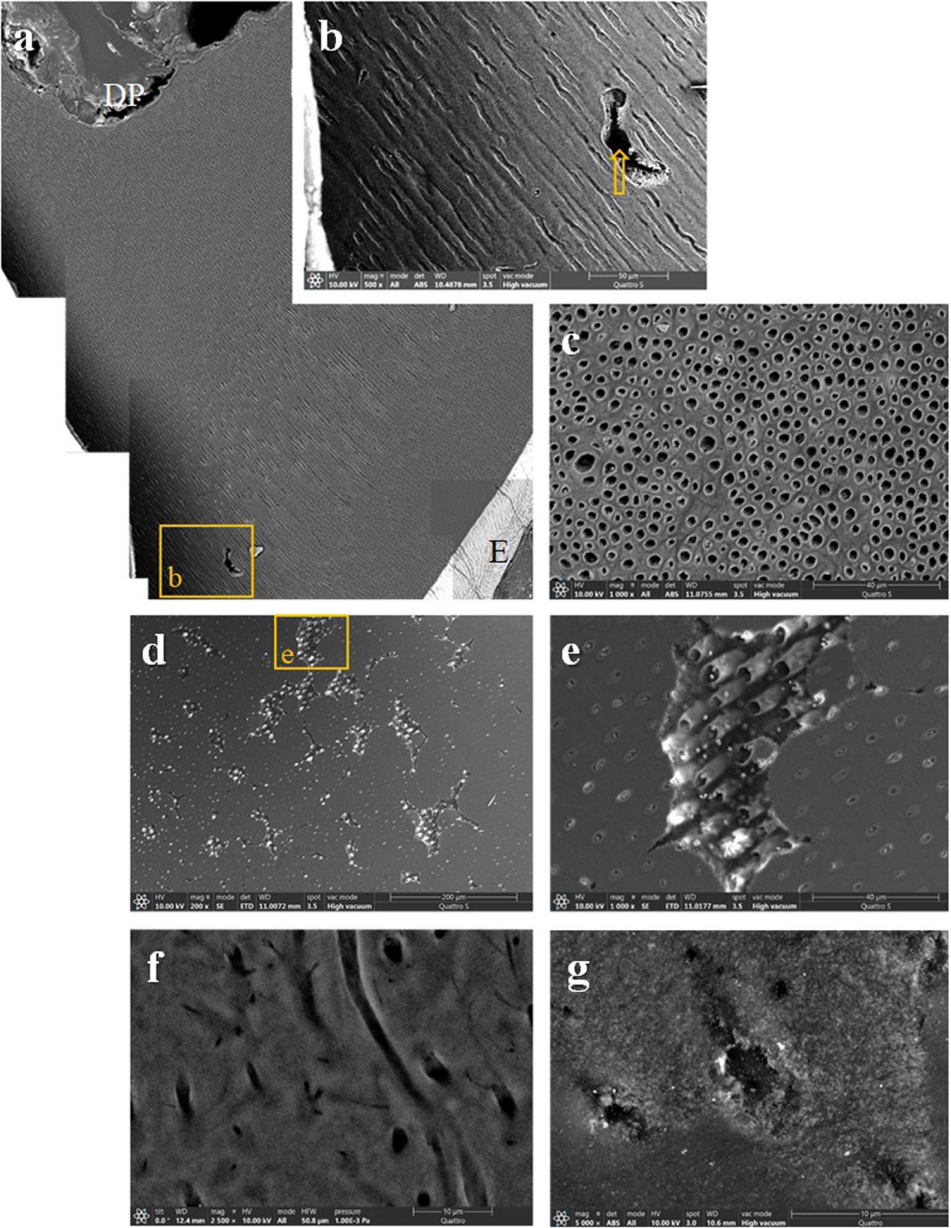
A crown fragment of a deciduous molar. **a** The compound image of an XLH-affected fragment of a deciduous molar (a tooth 65) shows the more severely affected dentin mineralisation in the outer half up to two-thirds of the thickness, with fluctuating tubules with wider lumen and also cracks in the enamel (200x, BSE and SE, unetched). **b** Under higher magnification, in the outer third of the dentin thickness, poorly distinctive peritubular dentin consisting of individual tubules with fluctuating diameters and less homogeneous greyish shading of the intertubular dentin, making this less mineralised dentin appear blurred. As in the previous specimen, the unusual void (arrow) can be seen (500x, BSE and SE, unetched). **c** Different diameters and shapes of transversely cut dentin tubules and a less homogeneous intertubular dentin structure indicate inadequate dentin structure and mineralisation (×1000, BSE and SE, unetched). **d** Interglobular dentin in an area of significantly poorer mineralisation shows plaques with indented areas (200x, SE, unetched) **e**, which are completely mineralised except for the thin peritubular dentin (×1000, SE, unetched). **f** At higher magnification, inhomogeneous intratubular dentin and a disordered course of the dentin tubules (×2500, BSE and SE, unetched) show the deficient dentin mineralisation. **g** Higher magnification shows inhomogeneous intertubular dentin with transversely cut dentin tubules of different diameters and shapes (×5000, BSE and SE, unetched). BSE backscattered electrons, E enamel, DP dental pulp, SE secondary electrons.

**Fig. 6:**
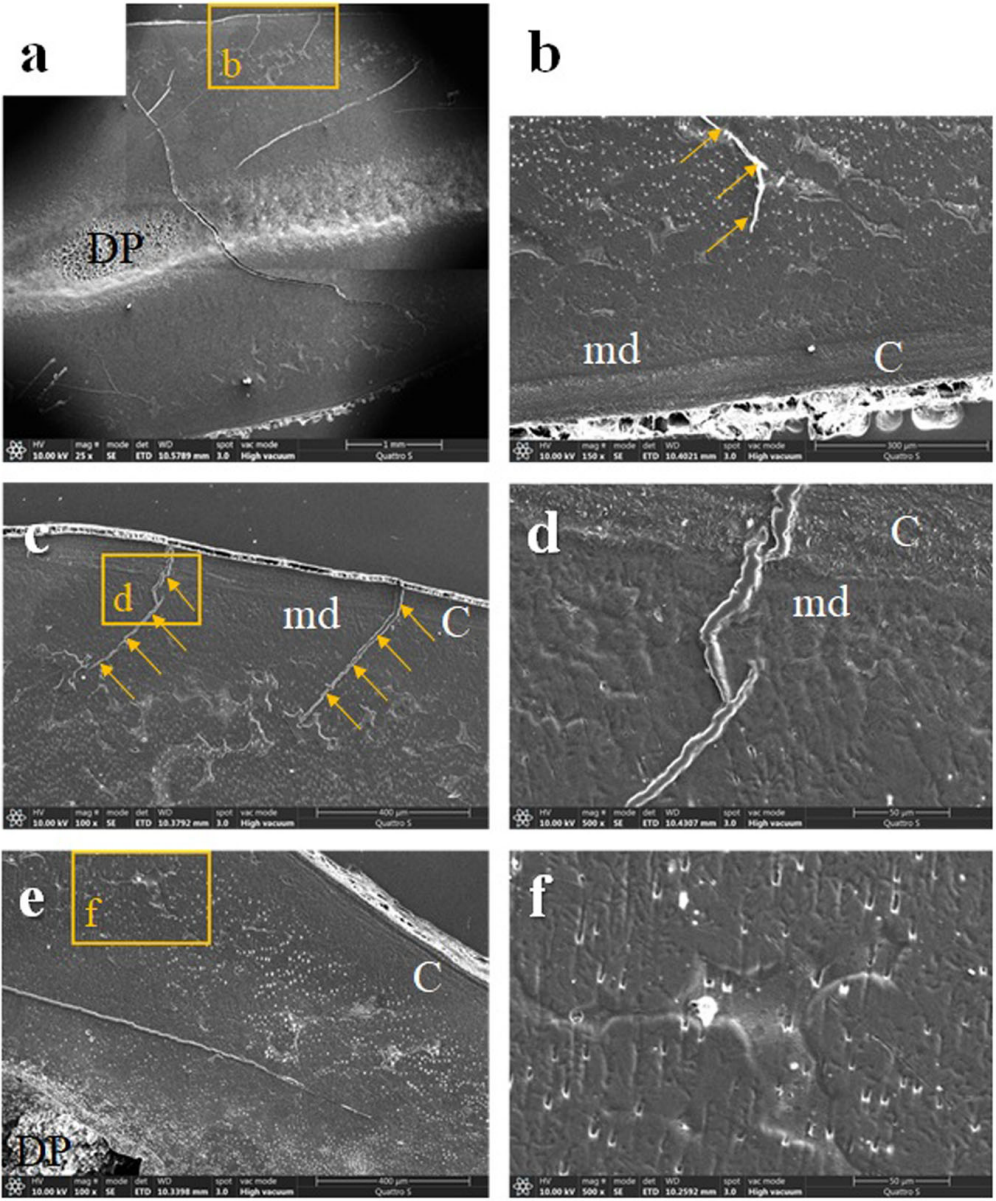
A supernumerary tooth. **a** A longitudinally sectioned supernumerary XLH-affected tooth shows areas of interglobular dentin that account for about half of the total dentin thickness. However, the cracks spreading across the root, which probably originate from the tooth extraction, also indicate inadequate mineralisation of the remaining dentin (compound image ×25, SE, etched). **b** Cracks propagate from the predentin towards the central part of the dentin thickness (arrows) (×150, SE, etched) or **c** from the root surface (arrows) through the cementum and the mantle dentin into the interglobular dentin (×100, SE, etched). **d** At higher magnification, a crack filled with epoxy can be seen extending from the cementum surface into the dentin (×500, SE, etched). **e** Under a thin layer of mantle dentin, interglobular dentin extends over half the root thickness. Note a crack (arrows) extending through the dentin (×100, SE, etched). **f** A concave hollow indentation is visible at higher magnification in the interglobular dentin (×500, SE, etched). BSE backscattered electrons; C cementum; DP dental pulp; md mantle dentin; SE secondary electrons.

Notably, less mineralised dentin was observed in all samples. In all four samples, the most severely affected dentin extended over approximately one-third to one-half of the outer dentin (Figs. 3, 4, 5, 6). Beneath the thin line of mantle dentin, the zone of inadequately mineralised interglobular dentin showed dentin tubules of uneven cross-section, size and distribution, with the peritubular dentin poorly differentiated from the intertubular dentin. Areas of indented dentin were visible in this zone, where dentin was completely absent to a certain depth, with the exception of thin peritubular dentin. Although mineralisation and structure were less abnormal in the remaining dentin, signs of pathologic aberration were evident elsewhere. Dentin tubules of uneven diameter coiled less orderly towards the predentin; mineralisation of intertubular and peritubular dentin was inadequate; in some places, peritubular dentin was indistinguishable from intertubular dentin.

The structure of the enamel appeared to be almost adequate; however, it was often interspersed with cracks through its thickness or chipped from the crown cusps (Figs. 3c, d, 4f, g, 5a), indicating deviations in its mineralisation. The cracks often continued from the enamel into the dentin. Cracks were also observed in the tooth’s root, either in the inner layer of the dentin or propagating from the root’s surface through the tooth cementum into the insufficiently mineralised dentin (Fig. 6).

### Literature search

To the best of our knowledge, there are 75 articles/case reports documenting XLH patients with verified periapical abscesses in deciduous and permanent teeth. The detailed case reports are presented in Table S1.

## Discussion

Clinical examination confirmed some abnormalities and characteristic pathological deviations in the oral cavity of all patients with XLH included in this study. In all patients, we observed a whitish to yellowish, milky glass-like colour of the dental crowns. Spontaneous periapical abscesses were observed in three male patients. Since these were also the oldest subjects in the study group and the likelihood of developing inflammation in teeth unaffected by caries, trauma, or periodontal disease increases with age, such findings were not unusual (Table S1). In addition, XLH disease is mainly caused by mutations in the *PHEX* gene on the X chromosome, leading to more severe phenotypes in males than in females.

During the SEM examination, we found considerable abnormalities in the dental tissues of three patients, especially in the dentin. In accordance with the literature, we identified large areas of interglobular spaces resulting from a lack of fusion of the calcospherites. We observed interglobular dentin in all specimens’ outer third to half of the dentin thickness. A similar observation of interglobular dentin in the outer part of the dentin of the teeth of XLH patients is reported by the Chaussain-Miller group [31] and by Opsahl Vital and coworkers [27]. Some other studies indicate that defective dentin is mainly present in the inner parts of the circumpulpal region [2] and with the increased width of the predentin [22]. An animal model (Hyp mice) confirmed the larger pulp chambers of teeth surrounded by wide predentin, thin dentin, and the presence of multiple interglobular dentin [32]. Comparable to the results of this study, where we observed human teeth from XLH patients, ultrastructural analysis of dentin from Hyp mice showed profound disorganisation of the peri- and intratubular structure, with odontoblast processes residing within the non-mineralised matrix sheath [2]. Similarly, we saw extensive cracks in the dentin, in the deciduous teeth and the supernumerary tooth. The cracks propagated through the central part of the tooth, from the root surface or enamel into the dentin, and were most likely caused by physiological occlusal forces or forces acting on the tooth during the extraction of insufficiently mineralised teeth.

The enamel was chipped off on the specimens’ crown cusps, presumably due to physiological biting forces. The enamel was repeatedly interspersed with cracks throughout its thickness, which often continued into the dentin. The results are consistent with descriptions of enamel in patients with XLH in the literature. Murayama and co-authors report cracks through the enamel [22], and Cremonesi and co-authors on deep microcracks on the enamel surface and an irregular enamel structure in hypomineralised teeth of patients with XLH [10]. The observed enamel changes could be due to insufficiently mineralised dentin supporting the enamel in the XLH patients. It is believed that the mantle dentin and the surface layer of circumpulpal dentin, with a total thickness of 250 µm, play a crucial role in the tooth’s biomechanical pressure resistance [33]. At the same time, it is reasonable to point out that most of the dentin is likely to be affected in patients with XLH, except the mantle dentin. The intactness of the mantle dentin is most likely reflected in less pronounced enamel defects and perhaps also in a somewhat slower progression of the bacteria towards the dental pulp. Why the mantle dentin remains largely intact is unclear. Perhaps this is due to the higher proportion of non-collagenous proteins of the organic matrix in the mantle dentin compared to the rest of the dentin.

The clinical and histological findings of the study confirmed an increased likelihood of dental inflammatory processes in patients with XLH. Less mineralised hard tissues and the cracks in these dental tissues allow oral bacteria to spread more easily and quickly. Unlike patients with vitamin D-dependent rickets or hypoparathyroidism, blood calcium levels are normal in patients with XLH. However, phosphorus deficiency in patients with XLH leads to impaired mineralisation of hard dental tissue, especially dentin [34]. The dentin of patients with XLH has a lower crystallinity and a higher carbon content than the dentin of control teeth [2]. Inadequate quantity and quality of hydroxyapatite crystals contribute significantly to the abnormalities in tooth development and the pathology of erupted teeth that are common in patients with XLH. In addition, impaired cementum formation contributes to an increased risk of periodontitis and frequent periapical processes [35, 36]

In most XLH patients, the aetiology is due to a mutation in the *PHEX* gene, with an X-linked mode of inheritance [37]. The phosphate-regulating endopeptidase (PHEX enzyme) produced by the *PHEX* gene is mainly expressed in bone (osteoblasts and osteocytes) and teeth (odontoblasts and cementoblasts) [1]. This enzyme is thought to be involved in regulating phosphate balance and FGF23 (translated by the *FGF23* gene) and in the cleavage of several other proteins [38]. The PHEX enzyme influences mineralisation by osteopontin (OPN), matrix-extracellular phosphoprotein (MEPE) and peptides rich in acidic serine and aspartate-rich motif (ASARM) [39–41]. Non-collagenous low molecular weight molecules formed in this way accumulate in the dentin of patients with hypophosphatemia, inhibit the fusion of calcospherites, and may act as local mineralisation inhibitors. It is unclear whether this is an active inhibitory process in the interglobular spaces or whether they are gradually distributed as waste molecules into non-mineralising spaces [25].

XLH can also be inherited as an autosomal dominant trait if there is a mutation in the *FGF23* gene or as an autosomal recessive trait if there is a mutation in the *DMP1*, *ENPP1* or *FAM20C* gene [4]. FGF23, produced by the *FGF23* gene, is important in bone mineralisation and vitamin D metabolism [42–44]. It is probably also directly involved in amelogenesis and dentinogenesis; the mRNA transcribed from the *FGF23* gene is detected in ameloblasts and odontoblasts [4, 45]. Some authors argue that the impaired tooth development in children with XLH is primarily influenced by FGF23-related mechanisms [4]. In contrast, others suggest it is due to a combination of hypophosphatemia, local extracellular matrix disorders and altered vitamin D metabolism [46].

We also observed malocclusion (treated or untreated) in all XLH patients. These findings are consistent with reports on the frequent presence of malocclusion in patients with XLH [1, 12, 13]. Insufficient bone development, manifested by smaller and/or inconsistent dental arches, is most likely the cause of crowding of the teeth and/or mesial occlusion (Class III) [47]. In addition, an open bite or impacted or ectopic eruption of the maxillary canines is frequently observed in patients with delayed growth of the maxilla compared to the mandible [48]. In some cases in this study, the timing of tooth eruption was also somewhat unusual. Similarly, delayed tooth development [49] and delayed eruption of deciduous and permanent teeth have been reported in the literature on patients with XLH [50].

In children with a family history of XLH, the disease is usually diagnosed shortly after birth [18]. In early childhood, a lower glomerular filtration rate can prevent excessive phosphate loss to a certain extent. Nevertheless, treatment of these patients should usually be started at 9 months. At 8-10 months, XLH patients with sporadic mutations also frequently develop rickets-like symptoms. In most cases, however, the disease is not recognised until two years of age [18], when the primary clinical manifestation, i.e. progressive bowing of the legs, becomes apparent [51]. Once the diagnosis is made, the standard treatment is to initiate a conventional treatment plan for XLH, which includes multiple daily oral intake of phosphate supplements and biologically active vitamin D (calcitriol or alfacalcidol) [52].

Regarding teeth, treatment with vitamin D and phosphates appears to prevent significant anomalies in tooth development, improve tooth mineralisation and reduce the risk of subsequent abscesses and severe periodontal disease [28, 31, 53]. XLH children born to XLH-affected mothers who received conventional treatment during pregnancy had fewer enamel and dentin defects compared to children with sporadic XLH [10]. The positive impact of hypophosphatemia treatment on oral health is particularly evident in those who started treatment in early childhood (i.e. during tooth development) and continued throughout [4]. Despite the proven beneficial effects of treatment with vitamin D and phosphates in patients with XLH, some authors believe that it generally does not prevent developmental defects of deciduous teeth [10] and only partially prevents permanent teeth’ dental and periodontal pathology [20]. Some authors argue that this treatment does not ensure normal phosphate levels and that many patients do not respond adequately or thoroughly to this therapy [8].

To a certain extent, the results of this study confirm such assumptions. The two oldest patients received the treatment described above from the tenth and twelfth month of life. Nevertheless, both showed oral signs and symptoms of XLH, including spontaneous periapical abscesses in permanent teeth. Lower patient compliance with treatment could contribute to such an outcome. Indeed, conventional treatment is particularly challenging for children, as it has gastrointestinal side effects and requires frequent dosing, parental supervision, and careful and repeated monitoring of the patient, which is necessary for appropriate dose adjustment [54]

On the other hand, conventional therapy does not target elevated FGF23 levels. On the contrary, it even inadvertently contributes to an increase in FGF23 levels, further impairs renal phosphate loss and reduces the efficacy of the treatment. In addition, long-term complications may develop with conventional treatment, including nephrocalcinosis, secondary/tertiary hyperparathyroidism and skeletal deformities due to inadequate control of rickets, especially in severe disease [55, 56].

In 2018, the possibility of a treatment based on the human monoclonal antibody against FGF23 was introduced [56]. This treatment with burosumab neutralises elevated FGF23 levels, resulting in better phosphate reabsorption in the renal tubules and phosphate absorption in the gastrointestinal tract, increased serum phosphate levels and the synthesis of endogenous 1,25(OH)2 vitamin D [8]. As a result, bone mineralisation is improved. An additional advantage is that the fortnightly administration of burosumab is more patient-friendly compared to multiple daily oral doses in conventional therapy [57].

While information on the positive effects of burosumab on children’s growth and biochemical profile has been accumulating since its introduction, there is a lack of data on its effects on dental health [58]. The effects on the potential prevention of periapical abscesses are particularly unclear [59]. While some authors report frequent periapical abscesses in patients treated with burosumab [60], others found a significantly lower incidence of periapical abscesses in the group of patients treated with burosumab compared to conventionally treated XLH patients [61]. Whether this is due to random patient variation or to burosumab remains unclear. It is known that the dental phenotype of XLH patients is not solely due to changes in FGF23, phosphate and vitamin D levels [62]. The lack of PHEX also leads to increased tissue peptide levels, such as osteopontin and MEPE, which remain unaffected by burosumab. This elevation may contribute to poor dentin mineralisation and, thus, increased susceptibility to periapical abscesses, independent of FGF23 or phosphate involvement [25, 41].

## Conclusion

XLH disease significantly affects the daily lives of these patients. Once the disease is diagnosed, patients must be treated for the rest of their lives. We found similar oral pathological signs and symptoms during the dental clinical examination. All patients also had malocclusions. The histology of the four tooth samples also confirmed similarities in the altered structure, especially of the dentin. The patients who donated teeth for the study (with complete primary dentition, early mixed dentition and complete permanent dentition) were treated conventionally from the age of 2.5, 3 and 1 year, respectively. The two youngest have been receiving burosumab since 2021. Based on the results of this study, patients with XLH appear to have an increased risk of some oral pathologies despite continuous XLH treatment. Further research is needed to understand better the impact of XLH disease and the different forms of treatment on dental mineralisation and the development of oral pathologies.

### Supplementary information

File S1

Table S1

Supplementary Legend

## Data Availability

The data supporting the findings of this study are available within the article and its supplementary materials.
