# Supplementary material for: Characteristics of oral health of patients with X-linked hypophosphatemia: case reports and literature review
Source: BDJ Open. 2024 May 31;10:42. doi: 10.1038/s41405-024-00223-6 (PMC11143263; doi:10.1038/s41405-024-00223-6)
Supplement: Supplementary file 1 — File S1 [file 41405_2024_223_MOESM1_ESM.pdf]

File S1. Search strategy

Electronic database (search completed on January 21, 2024)**:**

- PubMed:

1) rachitis AND abscess AND case report → 40

2) XLH AND abscess AND case report → 3

3) hypophosphatemic AND abscess AND case report → 19

- **Embase:**

1) rachitis.ti,ab. → 129

2) abscess.ti,ab. → 86436

3) "case report".ti,ab. → 564417

4) xlh.ti,ab. → 1152

5) hypophosphatemic.ti,ab. → 2882

6)1 or 4 or 5 → 3694

7) 2 and 3 and 6 → 3

- **Web of Science:**

1) (rachitis AND abscess AND case report) OR (XLH AND abscess AND case report) OR (hypophosphatemic AND abscess AND case report) → 12

- **Scopus:**

1) ALL ((rachitis AND abscess AND case AND report) OR (xlh AND abscess AND case AND report) OR (hypophosphatemic AND abscess AND case AND report)) → 202
