# Supplementary material for: Characteristics of oral health of patients with X-linked hypophosphatemia: case reports and literature review
Source: BDJ Open. 2024 May 31;10:42. doi: 10.1038/s41405-024-00223-6 (PMC11143263; doi:10.1038/s41405-024-00223-6)
Supplement: Supplementary file 2 — Table S1 [file 41405_2024_223_MOESM2_ESM.pdf]

Supplementary Table S1. Publications on XLH cases with spontaneous periapical abscesses.

Authors, Year			Number of patients included in the study	Patients with spontaneous periapical abscesses		
				No.	Age of patients (years)**	Abscessed tooth/teeth
1	Ainley, 1978	[1]	1	1	24	permanent
2	Alexander et al., 2001	[2]	1	1	16	deciduous and permanent
3	Andersen et al., 2012	[3]	52	*	5.7-74.5	permanent
4	Archard and Witkop, 1966	[4]	1	1	4.8	deciduous
5	Baroncelli et al., 2006	[5]	9	6	6.2-13.3	*
6	Batra et al., 2006	[6]	1	1	4.7	deciduous
7	Beltes and Zachou, 2012	[7]	1	1	15	deciduous and permanent
8	Bender and Naidorf, 1985	[8]	12	8	*	*
9	Boro et al., 2020	[9]	1	1	23	*
10	Boukpessi et al., 2017	[10]	7	5	5-17	deciduous and permanent
11	Bradley et al., 2021	[11]	2	2	16, 26	permanent
12	Brener et al., 2022	[12]	10	5	4.3 (mean)	deciduous
13	Chadwick and Aldred, 1992	[13]	1	1	6.6	deciduous
14	Chaussain-Miller et al., 2003	[14]	48	3	7, 10, 11	deciduous
15	Chaussain-Miller et al., 2007	[15]	7	4	3-16	deciduous and permanent

16	Clayton et al., 2021	[16]	6	2	5, 6	deciduous
17	Coelho et al., 2007	[17]	1	1	5	deciduous
18	Cohen and Becker, 1976	[18]	1	1	5	deciduous and permanent
19	Connor et al., 2015	[19]	52	52	39 (mean)	permanent
20	Cremonesi et al., 2014	[20]	10	2	8, 18	deciduous and permanent
21	Demirel et al., 2018	[21]	1	1	4.5	deciduous
22	Douyere et al., 2009	[22]	1	1	4	*
23	Friberg, 2013	[23]	3	2	43, 44	permanent
24	Gadion et al., 2022	[24]	71	29	*	*
25	Gallo and Merle, 1979	[25]	1	1	*	deciduous
26	Gao et al., 2018	[26]	2	1	5	deciduous
27	Gardner et al., 1969	[27]	2	2	*	deciduous and permanent
28	Gibson et al., 2022	[28]	7	4	*	deciduous and permanent
29	Godina and Belmont, 2013	[29]	1	1	4	deciduous
30	Goodman et al., 1998	[30]	17	11	2.6-11	deciduous and permanent
31	Gunther et al., 1943	[31]	1	1	3	deciduous
32	Hanisch et al., 2019	[32]	43	21	16-68	permanent
33	Harris and Sullivan, 1960	[33]	1	1	7.3	deciduous
34	Herbert, 1986	[34]	1	1	6	deciduous
35	Hughes and Hingston, 2019	[35]	1	1	3	deciduous
36	Imel et al., 2019	[36]	61	11	1-12	*
37	Jiajue et al., 2021	[37]	24	5	14-20	permanent

38	Jin et al., 2023 [38]	1	1	32	permanent
39	Kim et al., 2010 [39]	1	1	6	deciduous
40	Larsson et al., 2023 [40]	22	5 (+ one edentulous patient)	*	permanent
41	Lee et al., 2017 [41]	2	1	14	permanent
42	Marin et al., 2021 [42]	26	18	5-64	*
43	Marks et al., 1965 [43]	9	2	*	deciduous and permanent
44	McKee et al., 2013 [44]	2	1	6	deciduous
45	McWhorter and Seale, 1991 [45]	24	6	3-5.4	deciduous
46	Mohsenipour et al., 2017 [46]	19	2	*	*
47	Murayama et al., 2000 [47]	1	1	15	permanent
48	Nagarajappa et al., 2015 [48]	1	1	14	permanent
49	Paredes et al., 2018 [49]	1	1	7	deciduous
50	Pereira et al., 2004 [50]	3	1	19	permanent
51	Pliskin et al., 1975 [51]	1	1	*	permanent
52	Rabbani et al., 2012 [52]	19	2	*	*
53	Rakocz et al., 1982 [53]	1	1	5.5	deciduous
54	Rushton, 1959 [54]	1	1	21	permanent
55	Sandy et al., 2023 [55]	91	23	2 (mean)	*
56	Sauk and Witkop, 1973 [56]	1	1	4	deciduous
57	Schwartz et al., 1988 [57]	18	*	*	*
58	Seleme et al., 2022 [58]	1	1	31	permanent

59	Seow and Latham, 1986 [59]	13	6	7-32	deciduous and permanent
60	Seow et al., 1989 [60]	5	3	9, 12, 15	deciduous
61	Shroff et al., 2002 [61]	4	4	3.3-7.8	deciduous
62	Skrinar et al., 2019 [62]	322	235	1-74	deciduous and permanent
63	Soares et al., 2013 [63]	7	7	6-64	deciduous and permanent
64	Souza et al., 2010 [64]	14	1	*	*
65	Souza et al., 2013 [65]	1	1	5	deciduous
66	Stinton et al., 2016 [66]	1	1	14	permanent
67	Su et al., 2007 [67]	2	1	28	permanent
68	Tracy and Campbell, 1968 [68]	9	3	3.8, 10.3, *	deciduous and permanent
69	Tulloch and Andrews, 1983 [69]	3	3	*	deciduous and permanent
70	Vasilakis et al., 1980 [70]	1	1	*	permanent
71	Via, 1967 [71]	1	1	9	deciduous and permanent
72	Ward et al., 2022 [72]	61	11	3 patients <5, 8 patients ≥ 5 ***	*
73	Wato et al., 2020 [73]	1	1	1.4	deciduous
74	Whyte et al., 2019 [74]	13	7	1-4	deciduous
75	Wihr, 1970 [75]	1	1	*	deciduous

* - Not available.

** - In cases involving three patients or fewer, the ages of all patients are presented. For cases with four or more included patients, the age range is indicated. Additionally, in instances where the mean value was provided, it is clearly indicated in the table.

*** - Patients were divided into two groups: under five years old and five years and older.

References

1. Ainley JE Jr. Manifestations of familial hypophosphatemia. J Endod. 1978;4:26–8.
2. Alexander S, Moloney L, Kilpatrick N. Endodontic management of a patient with X-linked hypophosphataemic rickets. Aust Endod J. 2001;27:57–61.
3. Andersen MG, Beck-Nielsen SS, Haubek D, Hintze H, Gjørup H, Poulsen S. Periapical and endodontic status of permanent teeth in patients with hypophosphatemic rickets. J Oral Rehabil. 2012;39:144–50.
4. Archard HO, Witkop CJ. Hereditary hypophosphatemia (vitamin D-resistant rickets) presenting primary dental manifestations. Oral Surg. 1966;22:184–93.
5. Baroncelli GI, Angiolini M, Ninni E, Galli V, Saggese R, Giuca MR. Prevalence and pathogenesis of dental and periodontal lesions in children with X-linked hypophosphatemic rickets. Eur J Paediatr Dent. 2006;7:61–6.
6. Batra P, Tejani Z, Mars M. X-linked hypophosphatemia: dental and histologic findings. J Can Dent Assoc. 2006;72:69–72.
7. Beltes C, Zachou E. Endodontic management in a patient with vitamin D-resistant Rickets. J Endod. 2012;38:255–8.
8. Bender IB, Naidorf IJ. Dental observations in vitamin D-resistant rickets with special reference to periapical lesions. J Endod. 1985;11:514–20.
9. Boro H, Singh Naik S, Singh C, Khatiwada S, Khadgawat R. X-Linked Hypophosphatemic Rickets Manifesting as Sclerotic Bone Disease and Enthesopathy. Cureus. 2020;12:e10874.
10. Boukpessi T, Hoac B, Coyac BR, Leger T, Garcia C, Wicart P, et al. Osteopontin and the dento-osseous pathobiology of Xlinked hypophosphatemia. Bone. 2017;95:151–61.
11. Bradley H, Dutta A, Philpott R. Presentation and non-surgical endodontic treatment of two patients with X-linked hypophosphatemia: a case report. Int Endod J. 2021;54:1403–14.
12. Brener R, Zeitlin L, Lebenthal Y, Brener A. Dental health of pediatric patients with X-linked hypophosphatemia (XLH) after three years of burosumab therapy. Front Endocrinol (Lausanne). 2022;13:947814.

13. Chadwick BL, Aldred MJ. An unusual giant cell lesion in a child with vitamin D-resistant rickets. Int J Paediatr Dent. 1992;2:41–5.

14. Chaussain-Miller C, Sinding C, Wolikow M, Lasfargues JJ, Godeau G, Garabédian M. Dental abnormalities in patients with familial hypophosphatemic vitamin D-resistant rickets: prevention by early treatment with 1-hydroxyvitamin D. J Pediatr. 2003;142:324–31.

15. Chaussain-Miller C, Sinding C, Septier D, Wolikow M, Goldberg M, Garabédian M. Dentin structure in familial hypophosphatemic rickets: benefits of vitamin D and phosphate treatment. Oral Dis. 2007;13:482–9.

16. Clayton D, Chavez MB, Tan MH, Kolli TN, Giovani PA, Hammersmith KJ, et al. Mineralization defects in the primary dentition associated with X-linked hypophosphatemic rickets. JBMR. 2021;5:e10463.

17. Coelho A, Marques P, Canta JP. Case report: dental treatment of a child with hypophosphataemic rickets. Eur Arch Paediatr Dent. 2007;8:35–8.

18. Cohen S, Becker GL. Origin, diagnosis, and treatment of the dental manifestations of vitamin D-resistant rickets: review of the literature and report of case. J Am Dent Assoc. 1976;92:120–9.

19. Connor J, Olear EA, Insogna KL, Katz L, Baker S, Kaur R, et al. Conventional therapy in adults with X-linked hypophosphatemia: effects on enthesopathy and dental disease. J Clin Endocrinol Metab. 2015;100:3625–32.

20. Cremonesi I, Nucci C, D’Alessandro G, Alkhamis N, Marchionni S, Piana G. X-linked hypophosphatemic rickets: enamel abnormalities and oral clinical findings. Scanning. 2014;36:456–61.

21. Demirel A, Altuğ AT, Erdemli E, Tulga Öz. Dental management of hypophosphatemic vitamin D resistant rickets. J Pediatr Res. 2018;5:221-4.

22. Douyere D, Joseph C, Gaucher C, Chaussain C, Courson F. Familial hypophosphatemic vitamin D-resistant rickets-prevention of spontaneous dental abscesses on primary teeth: a case report. Oral Surg Oral Med Oral Pathol Oral Radio Endod. 2009;107:525–30.

23. Friberg B. Brånemark system implants and rare disorders: a report of six cases. Int J Periodontics Restor Dent. 2013;33:139–48.

24. Gadion M, Hervé A, Herrou J, Rothenbuhler A, Smail-Faugeron V, Courson F, et al. Burosumab and dental abscesses in children with X-linked hypophosphatemia. JBMR. 2022;6:e10672.

25. Gallo LG, Merle SG. Spontaneous dental abscesses in vitamin-D-resistant rickets: report of case. ASDC J Dent Child. 1979;46:327–9.

26. Gao Y, Wang ZM, Li XL. Analysis of 2 novel mutations of PHEX gene inducing X-linked dominant hypophosphatemia rickets in 2 families: Two case reports. Med (Baltim). 2018;97:e11453.

27. Gardner DE, Davis WB, Prescott GH. Hereditary hypophosphatemia. ASDC J Dent Child. 1969;36:199. passim

28. Gibson C, Mubeen S, Evans R. X-linked hypophosphatemic rickets: Orthodontic considerations and management. A case report. J Orthod. 2022;49:205–12.

29. Godina HG, Belmont LF. Dental characteristics of hypophosphatemic rickets. Case report. Rev Odont Mex. 2013;17:103–10.

30. Goodman JR, Gelbier MJ, Bennett JH, Winter GB. Dental problems associated with hypophosphataemic vitamin D resistant rickets. Int J Paediatr Dent. 1998;8:19–28.

31. Gunther L, Cohen ET, Cohn WE, Greenberg DM. Metabolism of bone salts in resistant rickets: report of a case, with balance and radioactive tracer studies. Am J Dis Child. 1943;66:517–27.

32. Hanisch M, Bohner L, Sabandal MMI, Kleinheinz J, Jung S. Oral symptoms and oral health-related quality of life of individuals with x-linked hypophosphatemia. Head Face Med. 2019;15:8.

33. Harris R, Sullivan HR. Dental sequelæ in deciduous dentition in vitamin D resistant rickets: case Report. Aust Dent J. 1960;5:200–3.

34. Herbert FL. Hereditary hypophosphatemia rickets: an important awareness for dentists. ASDC J Dent Child. 1986;53:223–6.

35. Hughes SL, Hingston EJ. Spontaneous dental abscesses in hereditary hypophosphataemic rickets: a preventive restorative approach in the primary dentition. Dent Update. 2019;46:1067–70.

36. Imel EA, Glorieux FH, Whyte MP, Munns CF, Ward LM, Nilsson O, et al. Burosumab versus conventional therapy in children with X-linked hypophosphataemia: a randomised, active-controlled, open-label, phase 3 trial. Lancet. 2019;393:2416–27.

37. Jiajue R, Ni X, Jin C, Huo L, Wu H, Liu Y, et al. Early discrimination between tumor-induced rickets/osteomalacia and xlinked hypophosphatemia in chinese children and adolescents: a retrospective case-control study. J Bone Min Res. 2021;36:1739–48.

38. Jin X, Xu Y, Liu W, Shi Z, Sun Y, Pan X, et al. Dental manifestations and treatment of hypophosphatemic rickets: A case report and review of literature. BDJ Open. 2023;9:2.

39. Kim SJ, Park JH, Kim KC, Choi S. VITAMIN D-RESISTANT RICKETS : A CASE REPORT. J Korean Dis Oral Health. 2010;6:10–4.

40. Larsson A, Regnstrand T, Skott P, Mäkitie O, Björnsdottir S, Garming-Legert K. Dental health of patients with X-linked hypophosphatemia: a controlled study. Front Oral Health. 2023;4:1087761.

41. Lee BN, Jung HY, Chang HS, Hwang YC, Oh WM. Dental management of patients with X-linked hypophosphatemia. Restor Dent Endod. 2017;42:146–51.

42. Marin A, Morales P, Jiménez M, Borja E, Ivanovic-Zuvic D, Collins MT, et al. Characterization of oral health status in chilean patients with X-linked hypophosphatemia. Calcif Tissue Int. 2021;109:132–8.

43. Marks SC, Lindahl RL, Bawden JW. Dental and cephalometric findings in vitamin D resistant rickets. J Dent Child.1965;32:259–65.

44. McKee MD, Hoac B, Addison WN, Barros NM, Millán JL, Chaussain C. Extracellular matrix mineralization in periodontal tissues: Noncollagenous matrix proteins, enzymes, and relationship to hypophosphatasia and X-linked hypophosphatemia. Periodontol 2000. 2013;63:102–22.

45. McWhorter AG, Seale NS. Prevalence of dental abscess in a population of children with vitamin D-resistant rickets. Pediatr Dent. 1991;13:91–6.

46. Mohsenipour R, Mohebbi A, Rostami P, Fallahi A, Rahmani P. Prevalence of dental abnormalities in different calcium metabolism disorders in a group of Iranian children. Biomed Res. 2017;28:6757–62.

47. Murayama T, Iwatsubo R, Akiyama S, Amano A, Morisaki I. Familial hypophosphatemic vitamin D-resistant rickets: dental findings and histologic study of teeth. Oral Surg Oral Med Oral Pathol Oral Radio Endod. 2000;90:310–6.

48. Nagarajappa AK, Sreedevi, Pandya D, Ravi KS. Case report grade II dental manifestations in rickets: a case report. Int J Med Appl Sci. 2015;4:146–50.

49. Paredes SEY, Segato RAB, Moreira LD, Moreira A, Serrano KVD, Rodrigues CT, et al. Dentoalveolar abscesses not associated with caries or trauma: a diagnostic hallmark of hypophosphatemic rickets initially misdiagnosed as hypochondroplasia. Head Neck Pathol. 2018;12:604–9.

50. Pereira CM, de Andrade CR, Vargas PA, Coletta RD, de Almeida OP, Lopes MA. Dental alterations associated with X-linked hypophosphatemic rickets. J Endod. 2004;30:241–5.

51. Pliskin ME, Brown AM, Baden EE, Kimball HG. Vitamin D resistant rickets of a young adult patient. A review and case report. J Oral Med. 1975;30:77–80.

52. Rabbani A, Rahmani P, Ziaee V, Ghodoosi S. Dental problems in hypophosphatemic rickets, a cross sectional study. Iran J Pediatr. 2012;22:531–4.

53. Rakocz M, Keating J 3rd, Johnson R. Management of the primary dentition in vitamin D-resistant rickets. Oral Surg Oral Med Oral Pathol. 1982;54:166–71.

54. Rushton MA. Two specimens illustrating proteolysis of dentine. Proc R Soc Med. 1959;52:115–7.

55. Sandy JL, Nunez C, Wheeler BJ, Jefferies C, Morris A, Siafarikas A, et al. Prevalence and characteristics of paediatric Xlinked hypophosphataemia in Australia and New Zealand: Results from the Australian and the New Zealand Paediatric Surveillance Units survey. Bone. 2023;173:116791.

56. Sauk JJ Jr, Witkop CJ Jr. Electron optic analysis of human dentin in hypophosphatemic vitamin D-resistant rickets (report of a kindred with consanguinity). J Oral Pathol. 1973;2:203–14.

57. Schwartz S, Scriver CR, Reade TM, Shields ED. Oral findings in patients with autosomal dominant hypophosphatemic bone disease and X-linked hypophosphatemia: further evidence that they are different diseases. Oral Surg Oral Med Oral Pathol. 1988;66:310–4.

58. Seleme CB, Murakami GJC, Lima MLG, Maciel JVB, De Lima AAS, De Araòjo MR, et al. Dental and endodontic management in a patient with familial x-linked hypophosphatemic rickets. Int J Odontostomat. 2022;16:81–7.

59. Seow WK, Latham SC. The spectrum of dental manifestations in vitamin D-resistant rickets: implications for management. Pediatr Dent. 1986;8:245–50.

60. Seow WK, Romaniuk K, Sclavos S. Micromorphologic features of dentin in vitamin D-resistant rickets: correlation with clinical grading of severity. Pediatr Dent. 1989;11:203–8.

61. Shroff DV, McWhorter AG, Seale NS. Evaluation of aggressive pulp therapy in a population of vitamin D-resistant rickets patients: a follow-up of 4 cases. Pediatr Dent. 2002;24:347–9.

62. Skrinar A, Dvorak-Ewell M, Evins A, Macica C, Linglart A, Imel EA, et al. The Lifelong Impact of X-Linked Hypophosphatemia: Results From a Burden of Disease Survey. J Endocr Soc. 2019;3:1321–34.

63. Soares EC, Costa FW, Ribeiro TR, Alves AP, Fonteles CS. Clinical approach in familial hypophosphatemic rickets: report of three generations. Spec Care Dentist. 2013;33:304-7.

64. Souza MA, Soares Junior LA, Santos MA, Vaisbich MH. Dental abnormalities and oral health in patients with Hypophosphatemic rickets. Clin (Sao Paulo). 2010;65:1023–6.

65. Souza AP, Kobayashi TY, Lourenço Neto N, Silva SM, Machado MA, Oliveira TM. Dental manifestations of patient with vitamin D-resistant rickets. J Appl Oral Sci. 2013;21:601–6.

66. Stinton NM, Uston KA, Davis CD. Hypophosphatemic rickets and pre-eruptive spontaneous dental abscess. J Dent Child (Chic). 2016;83:46–50.

67. Su JM, Li Y, Ye XW, Wu ZF. Oral findings of hypophosphatemic vitamin D-resistant rickets: report of two cases. Chin Med J (Engl). 2007;120:1468–70.

68. Tracy WE, Campbell RA. Dentofacial development in children with vitamin D resistant rickets. J Am Dent Assoc. 1968;76:1026–31.

69. Tulloch EN, Andrews FF. The association of dental abscesses with vitamin D resistant rickets. Br Dent J. 1983;154:136–8.

70. Vasilakis GJ, Nygaard VK, DiPalma DM. Vitamin D resistant rickets. A review and case report of an adolescent boy with a history of dental problems. J Oral Med. 1980;35:19 26.

71. Via WF Jr. “Spontaneous” degeneration of the dental pulp associated with phosphate diabetes. Oral Surg Oral Med Oral Pathol. 1967;24:623–8.

72. Ward LM, Glorieux FH, Whyte MP, Munns CF, Portale AA, Högler W, et al. Effect of burosumab compared with conventional therapy on younger vs older children with X-linked hypophosphatemia. J Clin Endocrinol Metab. 2022;107:e3241– e53.

73. Wato K, Okawa R, Matayoshi S, Ogaya Y, Nomura R, Nakano K. X-linked hypophosphatemia diagnosed after identification of dental symptoms. Pediatr Dent J. 2020;30:115–9.

74. Whyte MP, Carpenter TO, Gottesman GS, Mao M, Skrinar A, San Martin J, et al. Efficacy and safety of burosumab in children aged 1-4 years with X-linked hypophosphataemia: a multicentre, open-label, phase 2 trial. Lancet Diabetes Endocrinol. 2019;7:189–99.

75. Wihr NL. Abnormal dentition in vitamin D resistant rickets. A case report. ASDC J Dent Child. 1970;37:222–4.
