# Supplementary material for: Characteristics of oral health of patients with X-linked hypophosphatemia: case reports and literature review
Source: BDJ Open. 2024 May 31;10:42. doi: 10.1038/s41405-024-00223-6 (PMC11143263; doi:10.1038/s41405-024-00223-6)
Supplement: Supplementary file 3 — Supplementary Legend [file 41405_2024_223_MOESM3_ESM.docx]

File S1: A detailed search strategy for the literature review of XLH cases with spontaneous periapical abscess formation using four electronic databases (PubMed, Embase, Web of Science and Scopus). 2.

Table S1: Articles and case reports documenting XLH patients with verified periapical abscesses in deciduous and permanent teeth.
